# Virulence difference between the prototypic Schu S_4_ strain (A1a) and F*rancisella tularensis* A1a, A1b, A2 and type B strains in a murine model of infection

**DOI:** 10.1186/1471-2334-14-67

**Published:** 2014-02-06

**Authors:** Claudia R Molins, Mark J Delorey, Brook M Yockey, John W Young, John T Belisle, Martin E Schriefer, Jeannine M Petersen

**Affiliations:** 1 Division of Vector-Borne Diseases, Centers for Disease Control and Prevention, Bacterial Diseases Branch, 3156 Rampart Road, Fort Collins, CO 80521 USA; 2 Department of Microbiology, Immunology, and Pathology, Colorado State University, Fort Collins, CO 80523 USA

**Keywords:** Prototypic strains, Murine model, Fever, *Francisella tularensis*, Tularemia, Schu S_4_, A1a

## Abstract

**Background:**

The use of prototypic strains is common among laboratories studying infectious agents as it promotes consistency for data comparability among and between laboratories. Schu S_4_ is the prototypic virulent strain of *Francisella tularensis* and has been used extensively as such over the past six decades. Studies have demonstrated virulence differences among the two clinically relevant subspecies of *F. tularensis, tularensis* (type A) and *holarctica* (type B) and more recently between type A subpopulations (A1a, A1b and A2). Schu S_4_ belongs to the most virulent subspecies of *F. tularensis*, subspecies *tularensis*.

**Methods:**

In this study, we investigated the relative virulence of Schu S_4_ in comparison to A1a, A1b, A2 and type B strains using a temperature-based murine model of infection. Mice were inoculated intradermally and a hypothermic drop point was used as a surrogate for death. Survival curves and the length of temperature phases were compared for all infections. Bacterial burdens were also compared between the most virulent type A subpopulation, A1b, and Schu S_4_ at drop point.

**Results:**

Survival curve comparisons demonstrate that the Schu S_4_ strain used in this study resembles the virulence of type B strains, and is significantly less virulent than all other type A (A1a, A1b and A2) strains tested. Additionally, when bacterial burdens were compared between mice infected with Schu S_4_ or MA00-2987 (A1b) significantly higher burdens were present in the blood and spleen of mice infected with MA00-2987.

**Conclusions:**

The knowledge gained from using Schu S_4_ as a prototypic virulent strain has unquestionably advanced the field of tularemia research. The findings of this study, however, indicate that careful consideration of *F. tularensis* strain selection must occur when the overall virulence of the strain used could impact the outcome and interpretation of results.

## Background

Tularemia, a disease caused by the Gram-negative bacterium *Francisella tularensis*, was first confirmed as a human infection in 1913 in a 21 year-old meat cutter from Ohio who developed ulcerative conjunctivitis and lymphadenitis after handling infected meat [1]. Approximately 25 years later, Foshay isolated *F. tularensis* (previously named *Bacterium tularense*) strain Schu from a finger ulcer of a patient in Ohio [2]. Virulence studies conducted in various animal models showed that this strain (also referred to as strain Sm) was of high virulence with an LD_100_ in rabbits of 1–100 bacterial cells [3-5]. Additionally, time to death experiments in guinea pigs and mice revealed that animals infected with this strain died earlier than those infected with a type B strain by one day when infecting mice and 3 to 4 days when infecting guinea pigs [4]. In 1951, Eigelsbach *et al*. conducted a study using the Schu strain to determine if *F. tularensis* colony morphology correlated with pathogenicity and immunogenic properties [6]. This study revealed colony morphologies that varied in color (buff, blue or grey), texture (smooth or nonsmooth) and LD_100_ (range from 1–10 to avirulent). One colony type, designated Schu S_4,_ for smooth (S) variant clone #4, was described as a smooth blue colony with a watery consistency, and highly virulent in mice with an LD_100_ of 1–10 organisms. Schu S_4_ has subsequently served as the representative strain for pathogenic studies of *F. tularensis* subspecies *tularensis* (type A), the most virulent of the three *F. tularensis* subspecies, *tularensis* (type A), *holarctica* (type B) and *mediasiatica*. Given its extensive use as a prototypic type A strain, the complete genome for Schu 4 was sequenced in 2005 [7].

Within the last decade, it has become increasingly clear that type A strains do not represent a uniform population. Multi-locus variable number tandem repeat (MLVA), pulsed field gel electrophoresis (PFGE), whole genome single nucleotide polymorphism (SNP) and whole genome sequencing have all been used to demonstrate a major split into two subpopulations, A.I (A1) and A.II (A2) [8-13]. Additional PFGE analyses have further identified two A1 subpopulations, A1a and A1b [14]. Although type A strains are well documented to be more virulent than type B strains (LD_100_ in rabbits of 1–100 bacterial cells for type A strains as compared to an LD_100_ of 10^9^ for type B strains) [4,15], more recent studies have revealed differing levels of virulence between the type A subpopulations A1a, A1b and A2 [14,16,17]. An epidemiological analysis of culture-confirmed human tularemia in the United States showed that infections caused by A1b strains resulted in significantly higher mortality (24%) than infections caused by A1a (4%) or A2 (0%). Logistic regression analysis of A1b infections further indicated that this higher mortality rate was not linked to host characteristics, suggesting that A1b strains have an intrinsic characteristic which makes them more virulent than A1a or A2 strains [14]. Additionally, a virulence study using a murine model of infection found that mice infected with A1b died significantly earlier than mice infected with A1a or A2 [16]. These results correlate with epidemiological findings for type A infections in humans and demonstrate the utility of murine models for identifying virulence differences among *F. tularensis* type A subpopulations.

Schu S_4_ has been classified by PFGE and SNP analysis as a type A strain belonging to the A1a and A.I.Br.SCHU S4 subpopulations, respectively [13,14] indicating the prototypic type A strain does not fall within the PFGE group of type A strains with highest virulence. Previous analyses indicate mice are a suitable system to monitor differences in virulence if survival curves opposed to LD_50_ are analyzed [16-18]. Additionally, most studies requiring an animal model for the study of *F. tularensis* are conducted in mice due to their ease of use. We therefore investigated the relative virulence of Schu S_4_ in comparison to A1a, A1b, A2 and type B strains isolated from more recent human cases using a murine model of infection and generated survival curves based on subcutaneous temperature measurements [18]. Our results revealed that the Schu S_4_ strain used in this study most closely resembles the virulence of type B strains, and is less virulent than all other type A strains tested here; A1a, A1b, and A2 strains.

## Methods

### Strains and culture conditions

The Schu S_4_ strain tested here is available through BEI (catalog number NR-643) and was deposited by the Centers for Disease Control. Schu S_4_ was grown from frozen stocks (−70°C) on cysteine heart agar supplemented with 9% sheep blood (CHAB) at 35°C for 48 h, followed by subculture onto CHAB for 24 h at 35°C. A bacterial suspension for inoculations was prepared in sterile saline. Colony forming units (CFU) in the inoculum was verified by spotting 50 μl of the inoculum onto each quadrant of two CHAB quad plates (8 replicates total) and letting the plates dry without spreading. CFU were counted after 48–72 hours of growth at 35°C. The Schu S_4_ strain used in this study was typed using PFGE as previously described [11,14].

Additional *F. tularensis* strains (n = 8) (see Additional file 1), two A1a (MO02-4195 and OK01-2528), two A1b (MA00-2987 and MD00-2970), two A2 (WY96-3418 and NM99-1823), and two type B (KY99-3387 and MI00-1730) strains, used in this study were described previously [16,18]. Several of these strains are available through BEI (WY96-3418, MA00-2987 and KY99-3387, and their respective catalog numbers are NR-644, NR-645 and NR-647).

### Mice and experimental protocol

Specific pathogen-free female C57BL/6 J mice, (The Jackson Laboratory, Bar Harbor, ME) 8–9 weeks of age, were used. For Schu S_4_ infections, mice (n = 7) were anesthetized by inhalation of isoflurane to effect and infected intradermally with 10–20 CFU (50 μl) via the tail dermis. Control mice (n = 7) were inoculated with 50 μl of saline. Seven mice were infected with Schu S_4_ to ensure sufficient power to detect a scale shift in the fitted survival curve corresponding to a mean survival time of at least one day. The temperature of infected mice was monitored every 1–2 hours (minimum of 12 observations per day) until they reached their drop point. The drop point is the time at which a febrile mouse’s temperature first drops below the mean temperature of the normal (early afebrile) phase [18]. Therefore, the mean temperature of the normal phase was determined for each mouse and mice were euthanized once the drop point was observed. Mice were given food at libitum and an exercise wheel was provided in every cage. All animal procedures were approved by the Division of Vector-Borne Infectious Diseases Institutional Animal Care and Use Committee (protocol number 08–012) and performed in accordance with the guidelines on the care and use of laboratory animals [19]. All animal experiments with *F. tularensis* were conducted in ABL3 facilities. Data for mice infected with strains representing each of the four *F. tularensis* subpopulations (A1a, A1b, A2 and type B) was previously published [16,18]. Mice were infected with Schu S_4_ during Round 2 of the previously published experiments [16]. In Round 2 experiments, seven mice were infected with strains MA00-2987 (A1b), MO02-4195 (A1a), NM99-1823 (A2) and MI00-1730 (type B), in addition to the seven control mice and seven Schu S_4_ infected mice described here. An additional infection of seven mice with MA00-2987 was also performed and these mice were euthanized at drop point.

### Comparative virulence

Survival times for Schu S_4_ infected mice were modeled according to a Weibull distribution (allowing both the scale and shape parameters to differ by strain) using the drop point as a surrogate of death. Previous experiments demonstrated that statistical inference from a comparison of survival curves generated for *F. tularensis* strains based on drop point were the same as those obtained using observed time to death [18]. Additionally, drop point is an ethical experimental endpoint, as mice are still responsive to stimuli and taking food and water. Standard diagnostics including residual plots and goodness of fit tests were used to validate the model fits. The survival curve generated for Schu S_4_ infected mice was compared to survival curves previously generated for eight *F. tularensis* strains; two A1a, two A1b, two A2 and two type B [16,18]. Survival curves for *F. tularensis* A1a, A1b, A2 and type B infected mice were also fitted to drop points, although mice were allowed to expire in order to establish and validate the drop point temperature model [18]. Differences in the parameter estimates (shape and scale) of the survival curves were considered statistically significant if p < 0.008, with this level of significance determined using the Bonferroni adjustment for multiple comparisons.

Survival function parameters were simulated from the estimated parameter distributions. From these, 10,000 survival times were simulated and used to compute the probability that a mouse infected with one strain “fails” before a mouse infected with a second strain for each *F. tularensis* group (A1a, A1b, A2 and type B as well as Schu S_4_). This was done 1,000 times to create distributions for the estimated probabilities from which 95% confidence intervals were obtained.

Comparisons between the lengths of time spent in the normal and febrile phases by Schu S_4_ infected mice and mice infected with A1a, A1b, A2 and type B strains were performed using a weighted ANOVA followed by multiple comparisons using Tukey’s method with an overall Type I error of 0.05.

### Quantitative bacteriology and mouse and organ weights

Mice infected with Schu S_4_ (A1a) were euthanized at drop point and whole organs (spleen, liver and lungs) and blood were removed aseptically. Whole organs and blood were also taken from control, non-infected mice and from mice infected with strain MA00-2987 (A1b) and euthanized at drop point [16,18]. Each organ was weighed and sterile saline was added (5 ml to spleen and lungs and 10 ml to liver) prior to homogenization using a Stomacher 80 micro Biomaster (Seward, Bohemia, NY). Homogenized samples were serially diluted in sterile saline and aliquots (50 μl) were spotted in duplicate onto CHAB agar quadrant plates without spreading. Colonies were counted after 48–72 h of growth at 35°C. Whole blood was collected from the abdominal aorta and diluted into sterile saline and plated as described [16]. Bacterial load comparisons were performed using MANOVA with an overall Type I error rate of α = 0.05 and confidence intervals for Pearson’s correlation were computed using Fisher’s z-transformation. Mice were weighed prior to infection and at drop point.

## Results

### Virulence comparison between Schu S_4_ and other A1a strains

**Figure 1:**
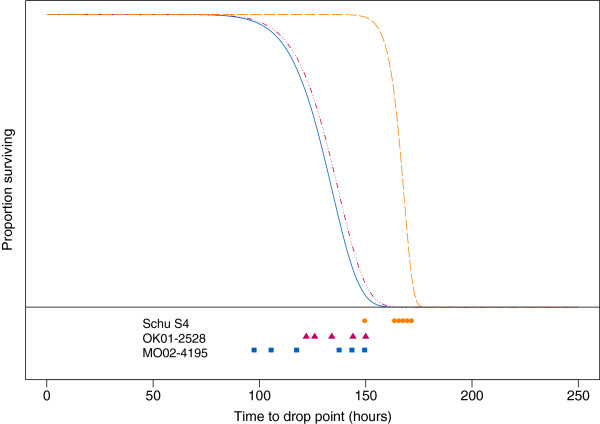
**Survival curve comparison of mice infected with A1a strains OK01-2528, MO02-4195, and Schu S**_**4**_**.** C57BL/6 J mice (n = 7/strain) were challenged intradermally with 10–20 CFU of *F. tularensis* A1a strains OK01-2528 (▬ **III** ▬; red), MO02-4195 (▬; blue) and Schu S_4_ (▬ ▬; orange) and survival curves were modeled according to a Weibull distribution. The drop point for each mouse is shown in orange circles for mice infected with strain Schu S_4_, red triangles for mice infected with OK01-2528 and blue squares for mice infected with MO02-4195.

The estimated shape (i.e. symmetry of pattern of time to drop point) and scale (i.e. mean and variability of time to drop point) parameters for the survival curve for mice infected with Schu S_4_ were compared to those from survival curves estimated previously for mice infected with two other A1a strains, OK01-2528 and MO02-4195 (Figure 1). Survival curves were generated using drop point as the surrogate for death. Drop point has been previously described and is the first temperature below the mean temperature of the normal phase [18]. Surprisingly, a significant difference (p < 0.008) was observed in both the scale and shape parameters for survival curves of mice infected with OK01-2528 or MO02-4195 compared to Schu S_4_. In comparison, as previously demonstrated, there was no difference (p > 0.008) between the scale and shape parameter for survival curves of mice infected with OK01-2528 and MO02-4195 [16,18]. The shift of the survival curve reflected that mice infected with Schu S_4_ reached drop point significantly later than mice infected with other A1a strains, while the shape of the curve indicated that mice infected with Schu S_4_ reached drop point within a significantly narrower timeframe as compared to mice infected with the two other A1a strains. As shown in Figure 1, mice infected with A1a strains OK01-2528 and MO02-4195 had all reached their drop point as mice infected with Schu S_4_ were beginning to reach drop point. Additionally, temperature measurements showed that the length of time in the normal phase (non-febrile, asymptomatic phase) was statistically longer for Schu S_4_ infected mice as compared to A1a infected mice by 27 hours (95% CI 12 to 42 hours) on average. Although the length of the febrile phase was not statistically different between Schu S_4_ and A1a infected mice, Schu S_4_ infected mice were in the febrile phase slightly longer (average of 47 hours) than A1a infected mice (average of 40 hours) [18]. At the time of infections, the average inoculation doses were determined to be similar among all three A1a strains, at 16 ± 3 CFU for OK01-2528, 11 ± 4 CFU for MO02-4195 and 14 ± 2 CFU for Schu S_4_, indicating that the significant differences observed for the Schu S_4_ survival curve were not directly attributable to dosage disparities [16].

### Virulence differences between *F. tularensis* strain Schu S_4_ and *F. tularensis* A1b, A2 and type B strains

**Figure 2:**
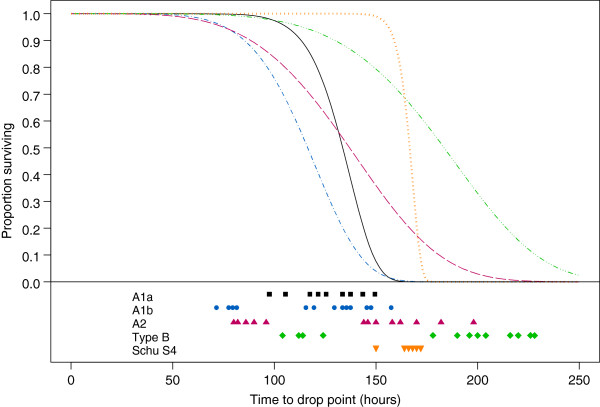
**Comparison of fitted survival curves for mice infected with *F. tularensis *A1a, A1b, A2, type B and Schu S**_**4**_**.** Survival curves were modeled previously [16,18] according to a Weibull distribution for mice (n = 7/strain) infected intradermally with 10–20 CFU of two strains each of A1a (▬; black), A1b (▬ **I** ▬; blue), A2 (▬ ▬; red), and type B (▬ **III** ▬; green), and are compared to the survival curve of Schu S_4_ infected mice (**III** ; orange). The time at which each mouse reached drop point is shown below the graph as black squares for A1a infected mice, blue circles for A1b infected mice, red triangles for A2 infected mice, green diamonds for type B infected mice and orange upside-down triangles for Schu S_4_ infected mice.

To determine if statistical differences in survival were also observed between Schu S_4_ and other type A as well as type B strains, the survival curve for Schu S_4_ infected mice was compared to survival curves for mice infected with *F. tularensis* A1b, A2 or type B strains (Figure 2) [16,18]. Two strains were used for each of the three *F. tularensis* groups; A1b, A2 and type B, with results combined to generate one survival curve per group. For reference, the combined survival curve for the two A1a strains from Figure 1 is shown (Figure 2).

Significant differences (p < 0.008) were observed in both the scale and shape parameters of the survival curve for Schu S_4_ infected mice when compared to the survival curves of mice infected with either A1b or A2 strains, and in the shape parameter when compared to the survival curve of mice infected with type B strains (Figure 2). There was no significant difference in the scale parameters between the survival curves of mice infected with Schu S_4_ as compared to type B strains (0.05 < p < 0.10). These data indicate that the mice infected with Schu S_4_ reached drop point significantly later than mice infected with A1b or A2 strains and that there was no statistical difference in the time to drop point between mice infected with Schu S_4_ as compared to type B strains. Additionally, as observed in the comparison with A1a strains, mice infected with Schu S_4_ reached drop point within a significantly narrower timeframe as compared to mice infected with A1b, A2 or type B strains.

Estimated survival distributions were used to determine whether mice infected with Schu S_4_ are likely to reach drop point after mice infected with A1a, A1b, A2 or type B strains (Table 1). The probability of a mouse infected with A1a, A1b or A2 strains reaching drop point before a mouse infected with Schu S_4_ was found to be 0.99, 0.99 and 0.79, respectively. This probability dropped to 0.33 for mice infected with type B strains.

**Table 1:** **Probability of A1a, A1b, A2 and type B infected mice reaching drop point before Schu S**_**4 **_**infected mice**

**Event**	**Probability**	**Lower 95% CI**^**a**^	**Upper 95% CI**
A1a versus Schu S_4_	0.99	0.95	1.00
A1b versus Schu S_4_	0.99	0.86	0.99
A2 versus Schu S_4_	0.79	0.59	0.93
Type B versus Schu S_4_	0.33	0.14	0.53

^a^CI: Confidence interval.

Differences in the length of time that mice spent in the normal and febrile phase of infection [18] revealed that the normal phase was statistically longer for mice infected with Schu S_4_ as compared to mice infected with A1b by an average of 42 hours (95% CI 18 to 65 hours). No statistical differences were observed between the normal phases of Schu S_4_, A2 and type B infected mice. When the length of time in the fever stage was compared, no statistical differences between Schu S_4_ and A1b, A2 or type B were observed. Similar to Schu S_4_ infected mice, the average time that type B infected mice were in the normal phase differed significantly from that of A1a and A1b infected mice. Type B infected mice were in the normal phase on average 47 hours (95% CI 15 to 80 hours) longer compared to A1a infected mice and on average 62 hours (95% CI 25 to 99 hours) longer compared to A1b infected mice.

### Bacterial load comparison between mice infected with A1a strain Schu S_4_ and A1b strain MA00-2987

**Figure 3:**
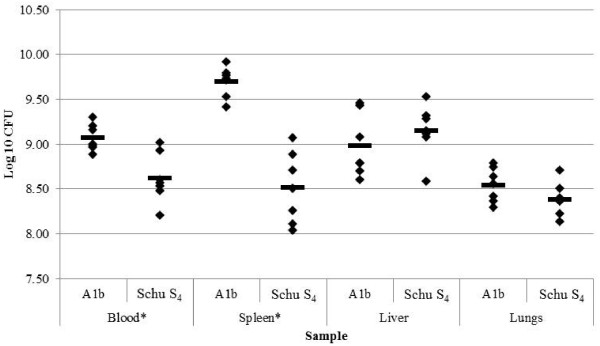
**Bacterial burden within the blood, spleen, liver and lungs of infected mice with A1a strain Schu S**_**4 **_**and A1b strain MA00-2987.** C57BL/6 J mice (n = 7/strain) were challenged intradermally with 10–20 CFU of *F. tularensis* strain MA00-2987 (A1b) and Schu S_4_ (A1a). When mice reached drop point, organs were harvested and blood was taken from each mouse. Significant differences in bacterial burden between the two infecting strains were calculated using MANOVA with an overall Type I error rate of α =0.05 and are designated with an asterisk. The mean bacterial burden is shown as a bar.

Because *F. tularensis* A1b strains are the most virulent among type A strains and Schu S_4_ is used as a prototypic virulent type A strain, the bacterial burden at drop point was quantified within the blood, spleen, liver and lungs of mice infected with Schu S_4_ (A1a) and MA00-2987 (A1b) (Figure 3). The mean bacterial burden was statistically higher in the blood and spleen (p = 0.004 and p < 0.0001, respectively) of mice infected with the A1b strain MA00-2987 as compared to mice infected with the A1a strain Schu S_4_. No statistical difference was observed between the bacterial burden in the lungs and liver of mice infected with these two strains (p = 0.21 and p = 0.72, respectively). Calculations to determine whether the bacterial burden within the blood and spleen of Schu S_4_ and MA00-2987 infected mice correlated to drop point revealed only a moderate correlation of 0.62 (95% CI 0.13 to 0.87) between the bacterial burden in the spleen and drop point in Schu S_4_ infected mice. When comparing mouse and organ weight between animals infected with MA00-2987 and Schu S_4_, no significant differences were found in both the weight at drop point (p = 0.58) and the weight of all organs (p = 0.10). The average inoculation doses were determined to be similar for mice infected with Schu S_4_ and MA00-2987 with inoculation doses of 14 ± 2 CFU and 14 ± 3 CFU, respectively.

## Discussion

Schu S_4_ serves as the prototypic type A strain for much of the current *F. tularensis* research being performed and is often classified as “fully virulent”. It has been distributed internationally among laboratories and the maintenance and propagation history among laboratories is unknown. Moreover, any laboratory adaptation this strain has undergone over the 60+ years since it was isolated, or genomic variation between Schu S_4_ strains of different laboratories is undetermined as only a single strain, Schu 4, has been sequenced at the whole genome level [7].

Our present data show that this particular Schu S_4_ strain is significantly less virulent than more recent clinical A1a strains as well as A1b and A2 strains. Beyond PFGE typing [14], limited studies have been performed to compare the virulence and genetic make-up between A1a strains; however, virulence differences between A1a and A1b strains have been reported [17]. Twine *et al.* previously demonstrated a significant difference in time to death for BALB/c mice infected intradermally or via aerosol with Schu S_4_ as compared to the type A strain, FSC033 [17]. FSC033 falls into the same canSNP group A.1.Br.001/002 as the two A1b strains tested here, MA00-2987 and MD00-2970. Specifically, mice infected with FSC033, expired significantly earlier than mice infected with Schu S_4_ (FSC237; *Francisella* Strain Collection, Swedish Defence Research Agency, Umea, Sweden). Thus, the difference in virulence between Schu S_4_ and other type A strains appears to be conserved among Schu S_4_ strains from different laboratories and within two mouse strains. The Schu S_4_ strain (FSC237) used in Twine *et al.* was the source of DNA for the published Schu S_4_ genome sequence. Twine *et al*. also reported unsuccessful attempts to enhance the virulence of Schu S_4_ by passage in mice; after five animal passages there was no change in the mean time to death for the Schu S_4_ strain. Together these results suggest that there is a fixed genetic basis responsible for the decreased virulence observed for Schu S_4_.

Surprisingly, the Schu S_4_ strain demonstrated a virulence phenotype with greater similarity to type B than to type A clinical strains. LD_50-__100_ experiments in rabbits and time to death experiments in mice or guinea pigs are lacking in the published literature for Schu S_4_, despite its use as a prototypic type A strain. *F. tularensis* Schu S_4_ was reported as being highly virulent based solely on an LD_100_ of 1–10 organisms in mice [6]. However, Bell and Olsufiev both demonstrated that virulence of *F. tularensis*, derived from LD_50_ determinations, in mice does not differentiate between type A and type B; both subspecies show an LD_50_ of 1 [4,20]. As no direct virulence comparisons (LD_50_ in rabbits or time to death differences in mice) have been reported in the literature, it is unknown if the virulence phenotype of the original Schu strain differs from that of Schu S_4_.

Schu S_4_ infected mice reached drop point in a significantly narrower timeframe as compared to mice infected with A1a, A1b, A2 and type B strains. Mice infected with A1a, A1b, A2 and type B consistently reached their drop point during two time frames as opposed to just one time frame as observed with Schu S_4_ infected mice. We hypothesize this is reflective of Schu S_4_ being a single colony selected from the original clinical isolate and laboratory adapted over time as compared to more recent clinical isolates with minimal laboratory manipulation. A few of the strains (recent clinical isolates) used in this study are also single colony picks. However, we tested whether this resulted in a selected phenotype by doing a side-by-side comparison of survival curves generated from mice infected with a single colony pick of the MA00-2987 strain and the original MA00-2987 isolate. There were no statistical differences between the two survival curves generated for these two infections (data not shown).

The mechanism for the difference in virulence of Schu S_4_ is unknown. However, our results indicate that bacterial burden at drop point was significantly higher in the blood and spleen of mice infected with the A1b strain, MA00-2987 as compared to mice infected with Schu S_4._ A similar observation was noted by Twine *et al.* who found that the FSC033 strain was better able to disseminate than Schu S_4_ from the original inoculation site to the spleen of infected mice [17]. Additionally, we previously showed that the bacterial burden in the blood and spleen at time to death did not differ between mice infected with A1a and A1b strains [16]. This suggests that the mechanism of bacterial dissemination may be altered in the Schu S_4_ strain as compared to MA00-2987 and other A1a strains. We also observed that Schu S_4_ infected mice remained in the normal phase (asymptomatic) significantly longer than mice infected with A1a or A1b clinical strains. Interestingly, type B infected mice which had a survival curve statistically indifferent (based on scale) from Schu S_4_ infected mice also displayed a longer normal phase length that was statistically different from those of A1a and A1b infected mice. Further studies are needed to better understand the virulence mechanisms of Schu S_4_ that differ from A1a and A1b strains and to determine how they compare to those of A2 and type B strains.

Overall, these findings indicate that infection of C57BL/6 J mice with Schu S_4_ does not completely mimic an infection with other type A strains. While the findings with Schu S_4_ in this study and in the Twine *et al.* study agree, it should be noted that the Schu S_4_ strain used here may not be equivalent to other Schu S_4_ strains distributed amongst various research institutions. Although the use of prototypic strains are important for consistency among research, the strain used should be appropriate for the work being performed and researchers need to acknowledge the limitations associated with this prototypic strain.

## Conclusions

In summary, we have evaluated the relative virulence of Schu S_4_ as compared to more recent A1a, A1b, A2 and type B clinical isolates of *F. tularensis* using a murine model*.* We demonstrated that Schu S_4_ is less virulent as compared to other type A strains and more closely resembles the virulence of type B strains. Schu S_4_ is still the prototypic pathogenic strain used and although this strain has a permanent role for such research, the observations from this study call into question the benefits of using more recent *F. tularensis* type A strains in addition to using the Schu S_4_ strain. The use of Schu S_4_ for studying *F. tularensis* has advantages, including the expediting of research because this strain is more readily available than non-prototypic strains, Schu S_4_ is well characterized and the data that results from the use of this strain can be directly compared between laboratories. However, the choice of Schu S_4_ as a “fully virulent” strain and as the sole type A challenge strain in murine models that evaluate the efficacy of human vaccine and therapeutics against *F. tularensis* infection, should be reconsidered.

## Abbreviations

CFU: Colony forming units; CHAB: Cysteine heart agar supplemented with 9% sheep blood; CI: Confidence interval; LD: Lethal dose; MLVA: Multi-locus variable number tandem repeat; PFGE: Pulsed field gel electrophoresis; SNP: Single nucleotide polymorphism.

## Competing interests

The authors declare that they do not have any competing interests.

## Author’s contributions

CRM performed the animal work, data analyses and drafted the manuscript. MJD performed the statistical analysis. BMY assisted with the mouse work and performed the culture work. JWY assisted in the design of the study and helped perform the mouse work. JTB assisted in the design of the study and helped draft the manuscript. MES assisted in the design of the study. JMP assisted in the design of the study and helped draft the manuscript. All authors read and approved the final manuscript.

## Pre-publication history

The pre-publication history for this paper can be accessed here:

http://www.biomedcentral.com/1471-2334/14/67/prepub

## Supplementary Material

Additional file 1 **This file contains a table that lists the *F. tularensis *** **strains used in this study.** Click here for file
